# Cancer following hip and knee arthroplasty: record linkage study

**DOI:** 10.1038/sj.bjc.6602511

**Published:** 2005-04-05

**Authors:** M J Goldacre, C J Wotton, V Seagroatt, D Yeates

**Affiliations:** 1 Unit of Health-Care Epidemiology, Department of Public Health, University of Oxford, Old Road Campus, Oxford OX3 7LF, UK

**Keywords:** arthroplasty, lymphoma, leukaemia, disease associations, record linkage

## Abstract

Concerns have been raised that degradation of implants used in hip and knee arthroplasty may lead to an increased risk of some cancers, particularly those of the haematopoietic, lymphatic and urinary systems. We used linked statistical records of hospital admissions and deaths to compare cancer rates in cohorts of people who had undergone hip or knee arthroplasty with a comparison cohort. We did not find an elevated risk for cancer, overall, in either the hip or knee cohort or in both combined (rate ratio for both combined 0.99; 95% confidence intervals 0.95–1.02), or for haematopoietic, lymphatic or urinary system cancers. There was also no elevation in risk of cancer more than 10 years after arthroplasty. Our findings add to the evidence that arthroplasty is safe in respect of cancer risk.

Hip and knee arthroplasties are now very common operations. It would be important, both from clinical and public health perspectives, if they increased the risk of cancer. There has been long-standing interest in whether prolonged contact between body tissue and plastic and metal prostheses does increase the risk of cancer. Corrosion and normal wear and tear of prostheses are known to liberate polyethylene and metallic particles (Sunderman, 1989; Jacobs *et al*, 1998; Urban *et al*, 2000). If these have any pathological effect, it is thought that the corrosion products are most likely to affect the haematopoietic, lymphatic and urinary systems. An early cohort study showed a significant long-term elevation of haematopoietic and lymphatic cancers after hip arthroplasty (Gillespie *et al*, 1988). It also showed a deficit of breast and colorectal cancer (Gillespie *et al*, 1988). Several studies of hip and knee arthroplasty have been published since then, summarised by Visuri *et al* (2003), and a meta-analysis has been published combining the data from six Nordic studies (Visuri *et al*, 2003). The meta-analysis reported a small but significant deficit of cancers, overall, with significant deficits of lung, laryngeal, stomach and colorectal cancers. The presumed explanation was that the deficits reflected aspects of ‘healthy lifestyle’ of people who eventually need joint replacement. The authors of the meta-analysis also reported that the combined data showed a small but significant elevation of endometrial cancer, prostate cancer and skin melanoma in people who had undergone joint replacement.

To add to the evidence on cancer following joint replacement, we used the Oxford record linkage study (ORLS) to determine rates of cancers in people after hip or knee arthroplasty.

## METHODS

### Population and data

The Oxford Record Linkage Study (ORLS) includes brief statistical abstracts of records of all hospital admissions (including day cases) in National Health Service (NHS) hospitals, and all deaths regardless of where they occurred, in defined populations within the former Oxford National Health Service Region from January 1963 to March 1999 (Goldacre *et al*, 2000). The hospital data were collected routinely in the NHS as the Oxford Regional Health Authority's hospital discharge statistics. The death data were derived from death certificates. Data collection covered part of one health district and its associated hospitals from 1963 (population 350 000), two districts from 1966 (population 850 000), six districts from 1975 (population 1.9 million) and all of the region's districts from 1987 (population 2.5 million). With the agreement of the Region's Data Protection Steering Group, the data for each individual were linked together routinely, as records accrued, as part of the region's health information system. The data are now anonymised and archived.

The hip and knee arthroplasty cohorts were obtained by identifying statistical records of individuals who had been admitted to NHS hospitals for these operations. A reference cohort was constructed by selecting records of admission for various other medical and surgical conditions (see footnote of Table 2). This is based on our ‘reference’ group of conditions that has been used in other studies of inter-relationships between diseases (Goldacre *et al*, 2000). We searched the database for any subsequent NHS hospital care for, or death from, cancers in these cohorts. We excluded from the analysis anyone in the arthroplasty and reference cohorts who had a previous record of cancer or who had cancer on the record of admission for the arthroplasty or reference condition. We considered that rates of cancer in the reference cohort would approximate those in the general population of the region, while allowing for migration in and out of it (data on migration of individuals were not available). Following the practice of others (Visuri *et al*, 2003), we excluded the first year of results to reduce the possibility that any of the patients who underwent the operations might have had undiagnosed cancer at the time of the surgery.

### Statistical methods

We calculated rates of cancer based on person-years at risk. We took ‘date of entry’ into each cohort as the date of first admission for hip or knee arthroplasty or reference condition, and ‘date of exit’ for the analysis of cancer as the date of the first record of cancer (if any occurred), death, or 31 March 1999, whichever was the earliest. We calculated rates for each cancer in each arthroplasty cohort and in the reference cohort, standardising the rates by age (in 5-year age groups), sex, calendar year of first recorded admission and district of residence, using the combined hip or knee arthroplasty and reference cohorts as the standard population. We then calculated the ratio of the standardised rate of occurrence of cancer in the hip or knee arthroplasty cohort relative to that in the reference cohort. The confidence interval for the rate ratio and *χ*^2^ statistics for its significance were calculated as described elsewhere (Breslow and Day, 1987). We took *P*<0.05 as the initial level of statistical significance but, for those comparisons without a prior hypothesis, we also adjusted the probability values using the Bonferroni correction to allow for multiple comparisons.

We studied the results for each condition in the reference cohort separately, as well as in combination, to ensure that no individual condition disproportionately influenced the ‘expected’ number of people with subsequent cancer.

## RESULTS

There were 33 691 patients in the hip arthroplasty cohort, 10 182 in the knee arthroplasty cohort and 475 555 in the reference (control) cohort. Table 1 summarises the age distribution of patients in both cohorts. Patients who entered the hip replacement cohort had an average age of 69 years and those who entered the knee replacement cohort had an average age of 67 years. The average periods of follow up were, respectively, 7.7 and 5.4 years.

### Hip arthroplasty

There was no elevation of the overall risk of cancer in people who had undergone hip arthroplasty (Table 2): the rate ratio for all cancers combined was 0.98 (95% confidence intervals 0.94–1.01). A statistically significant deficit of lung cancer was found: rate ratio 0.86 (95% confidence interval 0.78–0.95).

Considering the cancers for which there was a prior hypothesis about elevation of risk related to arthroplasty particles, we found that the rate ratios were not significantly different from one. The rate ratio for lymphoma was 1.01 (0.82–1.24), for leukaemia 0.94 (0.75–1.15), for bladder cancer 0.99 (0.86–1.15) and for renal cancer 0.93 (0.69–1.24). Considering other cancers where the published literature has suggested that there may be a different risk from that in the general population, there were no significant differences in our study for cancers of the body of uterus, prostate, malignant melanoma or other skin cancer (Table 2).

For cancers that were first recorded at least 10 years after hip arthroplasty, the overall rate ratio was significantly less than one (0.86: 752 observed cancers, 871 expected; 95% confidence intervals 0.80–0.92). The rate ratio for lung cancer was 0.64 (82 observed, 128 expected; 0.51–0.80) and there was no elevation of risk for lymphoma (0.87; 0.56–1.30), leukaemia (0.99; 0.67–1.41), bladder cancer (0.94; 0.70–1.22) or renal cancer (0.71; 0.34–1.30).

### Knee arthroplasty

There was no elevation of the overall risk of cancer in people who had undergone knee arthroplasty (Table 3): the rate ratio was 1.05 (0.97–1.14). That for cancer in people at least 10 years after knee arthroplasty was 1.07 (0.86–1.33). Of the 33 cancers studied, nonmelanoma skin cancer (rate ratio 1.35; *P*=0.011) and pancreatic cancer (rate ratio 1.76; *P*=0.0002) were found to be significantly high. The latter remained significant when the Bonferroni correction was used to adjust for multiple comparisons (*P*=0.007). There was a nonsignificant elevation of risk for lymphoma 10 or more years after knee arthroplasty (five observed, 2.6 expected; rate ratio 1.92: 0.62–4.49). There was no elevation of risk for leukaemia (0.80; 0.10–2.89).

## DISCUSSION

The strengths of our study are that it is large and population-based. Although confined to hospitalised patients, arthroplasty and the vast majority of the cancer outcomes are conditions for which people are admitted to hospital. A potential weakness is that we do not have data about either hospital care for or death of people who migrate out of the region. We have to assume that the arthroplasty cohorts and the reference cohort are acceptably comparable in respect of migration.

Our study has a number of other limitations. We do not have data about socioeconomic status or about lifestyle factors such as exercise, smoking and alcohol consumption. However, as we discuss below, the findings that we would seek to explain in terms of such confounding, if we could, are those that show numerically small deficits or excesses of cancers that tend to be lifestyle-related and not those that have been hypothesised to be caused by arthroplasty. The finding in our cohort that lung cancer occurred less often than expected after hip replacement was also noted in the meta-analysis by Visuri *et al* (2003) and in another Swedish record-linkage study (Paavolainen *et al*, 1999). It is likely that patients who undergo joint replacements have, typically, led more active than average lifestyles. They are therefore less likely than average to have been smokers. It has also been suggested that physical activity, independently of smoking, may decrease the risk of lung cancer in men (Lee *et al*, 1999). Furthermore, patients selected for an elective operation are assessed and deemed to be healthy enough to warrant operation: there may be a selection effect for healthy patients in the arthroplasty cohorts. In our study, nonmelanoma skin cancer was more common in the knee arthroplasty cohort than in the reference cohort. There is some consistency across studies in the finding of a positive association between arthroplasty and skin cancer (Nyrén *et al*, 1995; Signorello *et al*, 2001; Visuri *et al*, 2003). The most plausible explanation is that people who eventually need arthroplasty have typically been more active than average and have also typically spent more than average periods of time exposed to sunlight.

Associations found between joint replacement and cancer in some other studies – cancers of the uterus, prostate and kidney – were not found in our study. Our finding that cancer of the pancreas was associated with knee replacement has not been noted in other studies. We recognise that we made a wide range of comparisons, but, as the result for cancer of the pancreas was highly significant (Bonferroni corrected *P*-value 0.0066), this result may not be due to chance alone. This association needs to be confirmed or reputed elsewhere.

Suspicions about a possible causal association between joint replacements and cancer are largely concerned with the effects of metal implants (Sunderman, 1989; Jacobs *et al*, 1998; Urban *et al*, 2000). It has been suspected that minute, free-roaming particles, resulting from wear and tear, could have carcinogenic effects and, in particular, might increase the risk of lymphoma, leukaemia and cancer of the urinary tract. Our findings add to the accumulating evidence that arthroplasty is in fact safe in these respects, at least within a period of several years. However, longer follow-up than that currently available from the published studies would be prudent.

## Figures and Tables

**Table 1:** Number of people admitted to hospital for hip or knee arthroplasty in each age group

	**Hip arthroplasty**		**Knee arthroplasty**	
**Age groups (years)**	**No.**	**%**	**No.**	**%**
<40	676	2	176	1.7
40–49	1144	3.4	257	2.5
50–54	1468	4.4	341	3.3
55–59	2541	7.5	732	7.2
60–64	4152	12.3	1243	12.2
65–69	5280	15.7	1873	18.4
70–74	6041	17.9	2249	22.1
75–79	5642	16.7	1928	19
80–84	3979	11.8	1049	10.3
85+	2768	8.2	334	3.3

Total	33 691	100	10 182	100

**Table 2:** Occurrence of cancer in people who had undergone hip replacement at least a year before: number of people in the reference cohort^a^ with each cancer, observed and expected number of people with cancer in the hip replacement cohort, ratio of rates in the hip replacement cohort to that in the reference cohort, and 95% confidence intervals for the rate ratio

**Cancer (ICD code)^b^**	**Number in cohort**	**Person-years of follow-up^c^**	**Observed number in hip replacement**	**Expected number in hip replacement**	**Adjusted rate ratio^d^**	**95% confidence interval**
All cancers (140–208)	25 047	5 616 936	3015	3077	0.98	0.94–1.01
Upper gastrointestinal (140–141, 143–146, 148–149)	342	5 727 181	49	49.4	0.99	0.72–1.34
Salivary gland (142)	99	5 728 311	5	8.5	0.59	0.19–1.37
Nasopharynx (147)	47	5 729 341	3	4.1	0.73	0.15–2.14
Oesophagus (150)	875	5 728 584	128	111	1.15	0.96–1.37
Stomach (151)	1507	5 727 192	179	192	0.93	0.80–1.08
Colon (153)	1797	4 580 723	400	402	0.99	0.89–1.11
Rectum (154)	1040	4 583 626	207	226	0.89	0.76–1.04
Liver (155)	320	5 729 463	50	41.9	1.19	0.89–1.57
Pancreas (157)	903	5 728 968	138	130	1.06	0.89–1.25
Lung (162)	4360	5 725 374	417	487	0.86	0.78–0.95
Breast (174, 175)	2432	5 711 342	380	390	0.97	0.88–1.08
Cervix (180)	221	2 311 285	25	28.2	0.89	0.57–1.31
Uterus (182)	385	2 310 745	76	68.2	1.11	0.88–1.39
Ovary (183.0)	139	2 314 062	25	21.3	1.17	0.76–1.73
Prostate (185)	2435	3 407 301	264	247	1.07	0.93–1.19
Testis (186)	142	3 413 649	0	2.4	0.00	0–1.54
Kidney (189.0, 189.1)	515	5 727 480	47	50.3	0.93	0.69–1.24
Bladder (188)	1770	5 718 361	194	195	0.99	0.86–1.15
Malignant melanoma (172)	399	5 726 836	39	39.8	0.98	0.70–1.34
Other skin cancer (173)	1967	5 712 787	237	245	0.97	0.86–1.15
Brain (malignant) (191)	456	5 728 457	40	37.9	1.06	0.75–1.44
Other nervous system (192)	56	5 729 228	6	3.7	1.62	0.60–3.53
Thyroid (193)	111	5 728 176	13	8.3	1.57	0.83–2.68
Bone (170)	160	5 728 776	14	16.8	0.83	0.46–1.40
Lymphoma (200–202)	937	5 724 562	97	95.8	1.01	0.82–1.24
Non-Hodgkin's lymphoma (200, 202)	828	5 726 174	95	91.8	1.03	0.84–1.27
Hodgkin's disease (201)	164	5 727 694	13	8	1.63	0.87–2.78
Multiple myeloma (203)	473	5 728 484	79	65.1	1.21	0.96–1.51
Leukaemia (204–208)	834	5 727 030	91	97.2	0.94	0.75–1.15
Lymphoid leukaemia (204)	398	5 727 893	37	42.2	0.88	0.62–1.21
Myeloid leukaemia (205)	427	5 728 884	49	50.9	0.96	0.71–1.27
Brain (benign) (225)	255	5 725 456	16	19.4	0.82	0.47–1.34

a Conditions used in reference cohort, with Office of Population, Censuses and Surveys (OPCS) code edition 3 for operations and ICD9 code for diagnosis (with equivalent codes used for other coding editions): tonsillectomy/adenoidectomy (OPCS 230–236), cataract (ICD9 366), squint (ICD9 378), otitis externa, otitis media (ICD9 380–382), haemorrhoids (ICD9 455; excluded from reference cohort for analyses of colorectal cancers), varicose veins (ICD9 454), upper respiratory tract infections (ICD9 460–466), deflected septum, nasal polyp (ICD9 470–471), impacted tooth and other disorders of teeth (ICD9 520–521), inguinal hernia (ICD9 550; excluded from reference cohort for analyses of colorectal cancers), ingrowing toenail and other diseases of nail (ICD9 703), sebaceous cyst (ICD9 706.2), internal derangement of knee (ICD9 717), bunion (ICD9 727.1), selected fractures (ICD9 810–816, 823–826), dislocations, sprains and strains (ICD9 830–839, 840–848), superficial injury and contusion (ICD9 910–919, 920–924).

b ICD 9 codes for each cancer (equivalent codes were used for cases coded in ICD Revisions 7, 8 and 10.).

c Person-years of follow-up vary a little between cancers because of different dates of onset and therefore of ‘exit’ from the analysis.

d Adjusted for sex, age in 5-year bands, district of residence and time period in single calendar years.

**Table 3:** Occurrence of cancer in people who had undergone knee replacement at least a year before: number of people in the reference cohort^a^ with each cancer, observed and expected number of people with cancer in the knee replacement cohort, ratio of rates in the knee replacement cohort to that in the reference cohort, and 95% confidence intervals for the rate ratio

**Cancer (ICD code)^b^**	**Number in cohort**	**Person-years of follow-up^c^**	**Observed number in knee replacement**	**Expected number in knee replacement**	**Adjusted rate ratio^d^**	**95% confidence interval**
All cancers (140–208)	24 691	5 586 906	640	609	1.05	0.97–1.14
Upper gastrointestinal (140–141, 143–146, 148–149,)	373	5 695 396	8	9.9	0.81	0.35–1.61
Salivary gland (142)	98	5 696 463	0	1.7	0.00	0–2.17
Nasopharynx (147)	47	5 697 482	0	0.7	0.00	0–5.27
Oesophagus (150)	860	5 696 753	26	22.6	1.15	0.75–1.74
Stomach (151)	1480	5 695 381	23	31.7	0.73	0.46–1.09
Colon (153)	1751	4 548 060	60	67.7	0.89	0.67–1.14
Rectum (154)	1010	4 550 822	46	35.0	1.31	0.96–1.75
Liver (155)	316	5 697 605	14	8.6	1.63	0.89–2.73
Pancreas (157)	889	5 697 157	42	23.9	1.76	1.27–2.38
Lung (162)	4309	5 693 572	88	96.3	0.91	0.73–1.13
Breast (174, 175)	2375	5 679 995	84	79	1.06	0.85–1.32
Cervix (180)	216	2 284 790	3	5.6	0.54	0.11–1.57
Uterus (182)	373	2 284 294	15	14	1.07	0.60–1.77
Ovary (183.0)	137	2 287 535	10	7.3	1.37	0.66–2.52
Prostate (185)	2426	3 402 004	48	48.8	0.98	0.73–1.30
Testis (186)	142	3 408 318	0	0.5	0.00	0–7.38
Kidney (189.0, 189.1)	515	5 695 635	8	10.5	0.76	0.33–1.50
Bladder (188)	1756	5 686 618	46	40.4	1.14	0.83–1.52
Malignant melanoma (172)	399	5 695 011	6	8.6	0.70	0.26–1.52
Other skin cancer (173)	1949	5 681 235	72	53.4	1.35	1.05–1.70
Brain (malignant) (191)	447	5 696 603	8	8	1.00	0.43–1.97
Other nervous system (192)	56	5 697 390	0	0.8	0.00	0–4.61
Thyroid (193)	111	5 696 325	2	1.3	1.54	0.19–5.56
Bone (170)	162	5 696 908	2	3.6	0.56	0.07–2.01
Lymphoma (200–202)	932	5 692 728	27	20.2	1.34	0.88–1.94
Non-Hodgkin's lymphoma (200, 202)	824	5 694 332	24	19.8	1.21	0.78–1.80
Hodgkin's disease (201)	163	5 695 843	3	1.4	2.14	0.44–6.26
Multiple myeloma (203)	463	5 696 638	15	12.9	1.16	0.65–1.92
Leukaemia (204–208)	820	5 695 219	16	19.7	0.81	0.46–1.32
Lymphoid leukaemia (204)	393	5 696 069	7	9.3	0.75	0.30–1.55
Myeloid leukaemia (205)	420	5 697 041	10	10	1.00	0.48–1.84
Brain (benign) (225)	252	5 693 620	2	4.4	0.45	0.06–1.64

For reference conditions and methods of standardisation, see footnotes of Table 2.
